# Correction: Sexual violence against migrants and asylum seekers. The experience of the MSF clinic on Lesvos Island, Greece

**DOI:** 10.1371/journal.pone.0300860

**Published:** 2024-03-14

**Authors:** Rea A. Belanteri, Sven Gudmund Hinderaker, Ewan Wilkinson, Maria Episkopou, Collins Timire, Eva De Plecker, Mzwamdile Mabhala, Kudakwashe C. Takarinda, Rafael Van den Bergh

There are errors in the Funding and Competing Interests statements. The correct statements are:

**Funding:** The operational research teaching was funded by the United Kingdom’s Department for International Development (DFID) through The Union against Chronic Lung Disease and TB (DFID Project number 202506); La Fondation Veuve Emile Metz-Tesch supported open access publications costs. These funders had no role in study design, data collection and analysis, decision to publish, or preparation of the manuscript. Médecins Sans Frontières (MSF) provided support for this study in the form of salaries for RAB, RVdB, EDP, and ME. The specific roles of these authors are articulated in the ‘author contributions’ section. Research conducted by MSF employees is done so within the scope of their job specification. MSF was involved in study design, data collection, analysis, decision to publish and preparation of the manuscript.

**Competing Interests:** The authors have read the journal’s policy and have the following competing interests to declare: Médecins Sans Frontières (MSF) provided support in the form of salaries for RAB, RVdB, EDP, and ME. This does not alter our adherence to *PLOS ONE* policies on sharing data and materials. There are no patents, products in development, or marketed products associated with this research.
