# Supplementary material for: Sexual violence against migrants and asylum seekers. The experience of the MSF clinic on Lesvos Island, Greece
Source: PLoS One. 2020 Sep 17;15(9):e0239187. doi: 10.1371/journal.pone.0239187 (PMC7498098; doi:10.1371/journal.pone.0239187)
Supplement: Attachment — Submitted filename: PONE-D-19-20179_Review_22aug19.docx [file pone.0239187.s001.docx]

General comments:

-Very interesting topic, there is little known about forced migration to Greece, a country affected by a very interesting economic crisis. The authors have conveyed the magnitude of sexual violence and the devastating effect it has on displaced people. A valuable contribution of this manuscript is the information on male and female survivors of sexual abuse.

An important concept that needs to be clarified are the categories of the location of the sexual assault. The three categories are country of origin, in transit or in Lesvos. It is unclear how long someone may be ‘in transit’ for. Timing is an important part of this analysis that needs clarification. There is a variable called ‘interval incidence-care’ that ranges from less than 72h to over a year. It seems that the clinic only sees acute cases (less than 72h), however 91% of cases presented within 1 month to 1 year since the incident. This technical level of information needs to be considered in the methods section and in the analytic plan. Of importance, each variable should be carefully defined in the methods section. For example, there is discussion of PEP eligibility but not what the eligibility criteria is.

The final section on mental health services is interesting, however the manuscript is already filled with 5 tables on health services and detailed information on sexual violence. I would recommend removing the section on mental health or more clearly including it in the objective statement and analysis plan. It seems to me that the questions about volume of new referrals to the MSF clinic is different from the main line of inquiry related to sexual violence.

Currently, the manuscripts reads more like an organizational report and less like an academic article. Below is a list of recommendations intended to improve the scientific contribution of the manuscript and its readability to a wider audience.

General comments:

-Kindly use the term ‘survivor’ in place of ‘victim’

-The manuscript should be reviewed for typo’s

-Rephrase sentences to start with words and not numbers

Tables

-suppress cell sizes under 5 to protect confidentiality

-review for consistency in font size, decimals

-Review table titles for consistency

-Include the total n for each column in Tables 3 and 4

Abstract

-Please present the frequency (n) and percentages for each variable

-The conclusion focuses on accessing services and mental health care, however there are not estimates on mental health presented in the results

-In place of words in quotations (e.g. “safe”), state the issue more objectively

Introduction

-I would generally review the manuscript to ensure that the title, abstract and manuscript are all conveying the same message.

-The first three paragraphs of the introduction focus broadly on sexual violence and it’s consequences. While interesting, the most important contribution of this article is the problem of sexual violence in humanitarian settings, in which there is an extensive amount of literature that should be added to the introduction. The specific situation of Greece and migration through the Mediterranean Sea, Balkans or the surrounding geographic area is a critical area to include. The issues surrounding migration by water or to islands would add value to the introduction section and the literature in general.

-In the fourth paragraph, the distinction between people in ‘transit or containment’ compared to those in conflict or post-conflict is unclear. The term containment is often used in infectious disease and outbreaks scenarios. Please clarify the term as it is used in this article or consider replacing it.

-Please review the objective statement so that it clearly prepares the reader for the methods and results section. A lot of the methods and part of the results feature issues of mental health, however this is not covered in the introduction or the objectives. This should be reconciled.

Methods

-There are six paragraphs dedicated to describing the setting. Please pick out the key points that summarize the setting and how it specifically relates to the research objectives and study design. I recommend moving some of the ‘General setting’ section to the introduction and include academic references

-In the Study population and period section, please describe the inclusion criteria and exclusion criteria. Is the sample restricted to people in the Moria camp who accessed the clinic and self-disclosed as survivors of sexual violence? Is the Moria camp an UNHCR camp? These details will clarify the external generalizability of the results.

-All variables presented in the tables should be previously defined in the methods

-Some of the statistics described in the methods section are not presented in the results section or tables. For example, the statistical analysis section describes using chi-square tests, however p-values, significance levels or chi-square estimates are not presented in the tables or text. There is mention of ‘groups’ but the group comparisons are not defined. Perhaps in transit vs Lesvos camp? Please clarify. If this is one of the main objectives of the analysis, it should be clearly stated in the objective statement and the analysis plan should be able to address the objectives

-There are many cells with small cell sizes where chi-square tests are not the most appropriate statistical test. Small cell sizes below 5 should be suppressed to protect confidentiality.

Results

-Please include n(%) in the results section

-Use numeric estimates in place of descriptors such as ‘a higher proportion’ or ‘increased substantially’. This is particularly important if significance testing was not performed. Keep the results section to describe the statistics and put the interpretations into the discussion section

In the ‘Specific setting’ section it says that the Moria camp provides services for those who presented for care less than 120 hours following the assault. However, in the results it says that 81% experienced sexual violence between 1-12 months before getting care. Please review.

Table 4 as described in the text says ‘Incidents tended to be more violent in the country of origin, with higher proportions of armed perpetrators and with more associated violence’ - This has not be statistically evaluated

Figure 1 is unclear

- ‘List Closed’ is an unclear category

Discussion

-Please clarify the issue with unofficial ‘migration professionals’ in paragraph three of the discussion

-Justify conclusions based on the shown results, avoid anecdotal information

-In the discussion of known perpetrators, please related the finding to comparable populations. The study of police reported sexual assault cases in the UK is not an appropriate reference.

Limitations section

-Include description of the measurement bias, selection bias

References

-Review for typos and inconsistent formatting
