# Supplementary material for: Sexual violence against migrants and asylum seekers. The experience of the MSF clinic on Lesvos Island, Greece
Source: PLoS One. 2020 Sep 17;15(9):e0239187. doi: 10.1371/journal.pone.0239187 (PMC7498098; doi:10.1371/journal.pone.0239187)
Supplement: Attachment — Submitted filename: Response to Reviewers.doc [file pone.0239187.s003.doc]

To the editor, PLOS ONE

Dear editor,

Thank you for your message including the reviews of our paper. We have amended the paper following the comments, and we think the paper is better now. In our responses below we refer to line numbers in the new revised version with track changes. Reviewers’ comments are shown, and our response is given point by point in **BOLD.**

Response to Reviewers

Reviewer #2: Thank you for the attentive revision. I am sincerely looking forward to seeing this piece in print. Article needs one more close proofread, however, as several typos remain (for example in lines 111, 217, 246).

**Thank you for your comments. All necessary changes are made accordingly.**

Reviewer #3: This topic is certainly timely and the work that the organization has done is admirable. But what this paper presents is less than a scholarly article and reads more like a research report -- and even then a somewhat limited one. There is a descriptive overview of this particular case and context but there is little that is drawn in terms of conclusions or steps for either corrective action, intervention or even suggestions for future study. There were many important findings that were mentioned in passing in the paper but not ever really followed up on. For example, the data suggests significant variance in the gender composition of survivors from what we commonly know, but this is not explored in greater detail. The percentage of survivors who were actually raped is also not commented upon other than briefly. The implication that so-called 'safe' countries are not safe at all was another avenue not followed. These are but a few. I am thus unable to recommend publication even though the topic is significant and it appears that the authors have already gone to some length to address earlier reviews.

**Thank you.**

**We recognise the limitations of the research. Thus, this is mentioned in “Discussion”, lines 346-355.**

**All necessary changes from native English co-author are done to improve the language issues found in the manuscript.**
